# Supplementary material for: Electrical analogue of one-dimensional and quasi-one-dimensional Aubry–André–Harper lattices
Source: Sci Rep. 2023 Aug 21;13:13633. doi: 10.1038/s41598-023-40690-9 (PMC10442325; doi:10.1038/s41598-023-40690-9)
Supplement: Supplementary file 1 — Supplementary Information. [file 41598_2023_40690_MOESM1_ESM.pdf]

Supplemental material for Electrical analogue of one-dimensional and quasi-one-dimensional Aubry-André-Harper lattices

Sudin Ganguly[1] and Santanu K. Maiti[2]

[1]Department of Physics, School of Applied Sciences, University of Science and Technology Meghalaya, Ri-Bhoi-793101, India

[2]Physics and Applied Mathematics Unit, Indian Statistical Institute, 203 Barrackpore Trunk Road, Kolkata-700108, India

August 16, 2023

1 One-to-one mapping between admittance matrix and TB Hamiltonian

To establish the analogy between the admittance matrix and the TBHamiltonian, we consider a 4-node circuit for the sake of simplicity, as depicted in Fig. 1(b) in the manuscript (for 8-node), which serves as an analogue to the 4-site 1D tight-binding chain shown in Fig. 1(a) in the manuscript (for 8 sites). Here we consider the nearest-neighbor scenario both in the circuit as well as for the TB chain.

By the Kirchhoff's current law, the net current flowing through any node is zero. Therefore, for the chosen 4-node circuit, we have four equations as follows:

$$\text{Node 1:} \quad j\omega C_N (V_1 - V_2) + j\omega C_1 V_1 + j\omega C_{\text{offset}} V_1 + \frac{V_1}{j\omega L_1} = 0 \tag{1}$$

$$\text{Node 2:} \quad j\omega C_N (V_2 - V_1) + j\omega C_N (V_2 - V_3) + j\omega C_2 V_2 + j\omega C_{\text{offset}} V_2 + \frac{V_2}{j\omega L_2} = 0 \tag{2}$$

$$\text{Node 3:} \quad j\omega C_N (V_3 - V_2) + j\omega C_N (V_3 - V_4) + j\omega C_3 V_3 + j\omega C_{\text{offset}} V_3 + \frac{V_3}{j\omega L_3} = 0 \tag{3}$$

$$\text{Node 4:} \quad j\omega C_N (V_4 - V_3) + j\omega C_4 V_4 + j\omega C_{\text{offset}} V_4 + \frac{V_4}{j\omega L_4} = 0 \tag{4}$$

Now, the admittance matrix can easily be constructed from the above equations, which reads

as

$$
\begin{aligned}
J_{mn} &= \begin{pmatrix} j\omega\left(C_N + C_1 + C_{\text{offset}}\right) + \frac{1}{j\omega L_1} & -j\omega C_N & 0 & 0 \\ -j\omega C_N & j\omega\left(2C_N + C_2 + C_{\text{offset}}\right) + \frac{1}{j\omega L_2} & -j\omega C_N & 0 \\ 0 & -j\omega C_N & j\omega\left(2C_N + C_3 + C_{\text{offset}}\right) + \frac{1}{j\omega L_3} & -j\omega C_N \\ 0 & 0 & -j\omega C_N & j\omega\left(C_N + C_4 + C_{\text{offset}}\right) + \frac{1}{j\omega L_4} \end{pmatrix} \\
&= j\omega \begin{pmatrix} \left(C_N + C_1 + C_{\text{offset}} - \frac{1}{\omega^2 L_1}\right) & -C_N & 0 & 0 \\ -C_N & \left(2C_N + C_2 + C_{\text{offset}} - \frac{1}{\omega^2 L_2}\right) & -C_N & 0 \\ 0 & -C_N & \left(2C_N + C_3 + C_{\text{offset}} - \frac{1}{\omega^2 L_3}\right) & -C_N \\ 0 & 0 & -C_N & \left(C_N + C_4 + C_{\text{offset}} - \frac{1}{\omega^2 L_4}\right) \end{pmatrix} \quad (5)
\end{aligned}
$$

Here ω is the driving frequency. V_n is the voltage at node n. C_n, and L_n are the grounded capacitor and inductor at node n, respectively.

Now, consider $L_2 = L_3 = L$ and $L_1 = L_4 = 2L$ and setting the driving frequency $\omega = 1/\sqrt{2LC_N}$. With these, the final form of the admittance matrix becomes

$$
J_{mn} = j\omega \begin{pmatrix} \left(C_1 + C_{\text{offset}}\right) & -C_N & 0 & 0 \\ -C_N & \left(C_2 + C_{\text{offset}}\right) & -C_N & 0 \\ 0 & -C_N & \left(C_3 + C_{\text{offset}}\right) & -C_N \\ 0 & 0 & -C_N & \left(C_4 + C_{\text{offset}}\right). \end{pmatrix} \quad (6)
$$

The structure of the admittance matrix is exactly same as the tight-binding Hamiltonian matrix of a 4-site 1D NN chain, which is

$$
H = \begin{pmatrix} \epsilon_1 & t_1 & 0 & 0 \\ t_1 & \epsilon_2 & t_1 & 0 \\ 0 & t_1 & \epsilon_3 & t_1 \\ 0 & 0 & t_1 & \epsilon_4 \end{pmatrix}. \quad (7)
$$

Comparing Eqs. 6 and 7, one can identify the onsite terms ϵ_n to the diagonal elements of the admittance matrix $j\omega\left(C_n + C_{\text{offset}}\right)$ and the nearest-neighbor hopping term t_1 with $-j\omega C_N$. The above formalism can be easily extended to any number of nodes and also for higher order connection scenarios.

Incorporation of AAH: The onsite potential modifies according to AAH model as expressed in Eq. 2 in the main text. Considering the parameters $b = (\sqrt{5}-1)/2$, $\phi_\nu = 0$, and $W = 1$, we can calculate the onsite energies as follows: $\epsilon_1 = -0.7374$, $\epsilon_2 = 0.0874$, $\epsilon_3 = 0.6084$, and $\epsilon_4 = -0.9847$. To align the circuit with these onsite values, we set the capacitors as $C_1 = -0.7374\,\mu$F, $C_2 = 0.0874\,\mu$F, $C_3 = 0.6084\,\mu$F, and $C_4 = -0.9847\,\mu$F. It is important to note that obtaining negative capacitance values can be achieved with the appropriate inductive coupling [1]. However, in the present case, it is not strictly necessary, as we are connecting the capacitor C_{offset} in parallel with C_n between each node and ground. Consequently, by appropriately selecting the value of C_{offset}, the net capacitance between each node and ground is always positive.

2 Concept of localization in correlated disordered circuit

The localization behavior in the circuit is determined based on the relative values of TPI. To analyze the behavior of TPI as a function of node index at different disorder strengths for the 1D NN circuit, we refer to Fig. 1. In Figs. 1(a), (b), and (c), we present the results specifically for the 1st and 52nd, and 100th eigenmodes, respectively, when the disorder strength W is set to zero. For computing the TPI, one node is fixed at the 1st node. In Figs. 1(a) and (b), we observe that the TPI reaches to a maximum value of approximately 0.015, whereas that in Fig. 1(c) is about 0.008. This value corresponds to the extent of participation of the eigenmode across the nodes in the circuit. Notably, the TPI exhibits oscillating behavior across the nodes, which is commonly observed in the local probability density or the local inverse participation ratio (IPR) behavior of a pristine 1D tight-binding chain. To investigate the behavior of the TPI with node index at

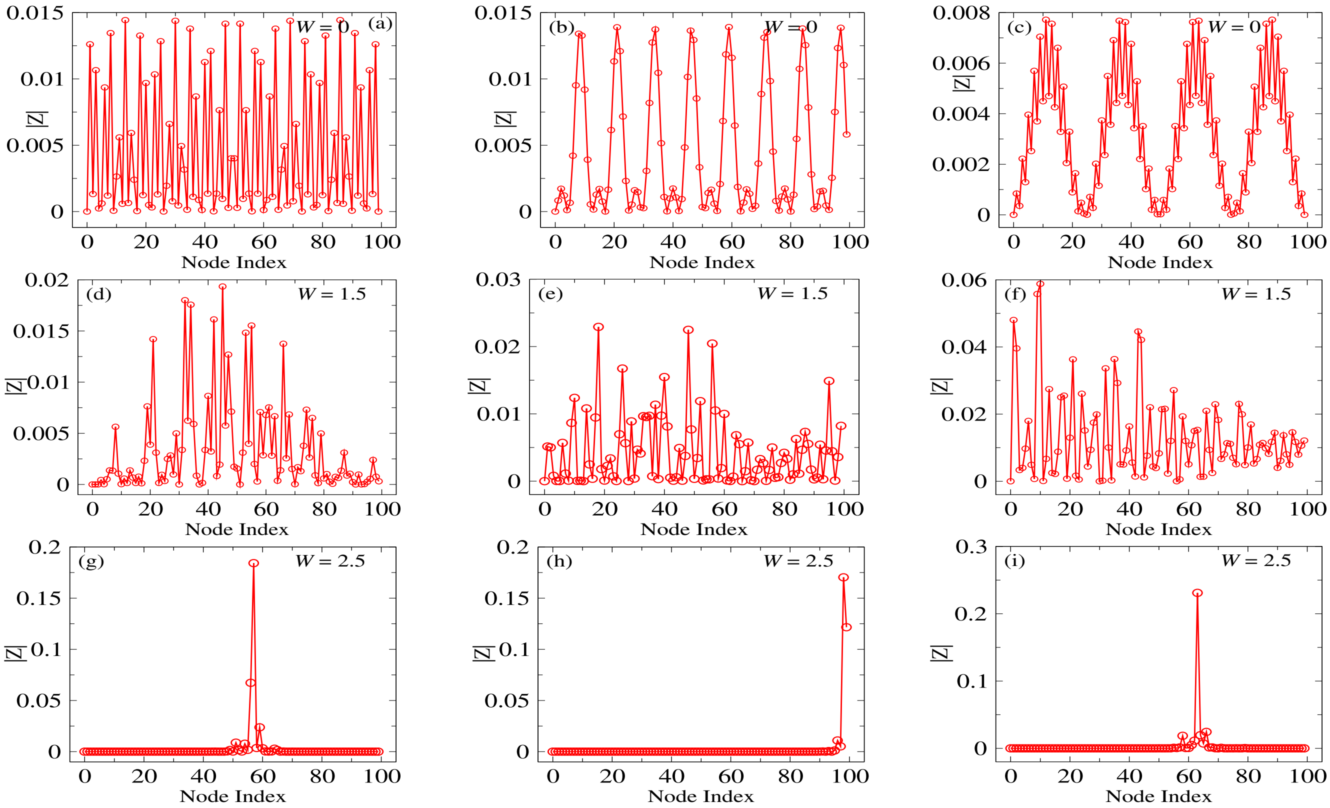

Figure 1: Two-port impedance $|Z|$ as a function of node index at different disorder strengths for the pth eigenmode. $W = 0$ for (a) 1st, (b) 52nd, and (c) 100th eigenmodes. $W = 1.5$ for (d) 1st, (e) 52nd, and (f) 100th eigenmodes. $W = 2.5$ for (g) 1st, (h) 52nd, and (i) 100th eigenmodes. For computing $|Z|$, one node is fixed at node 1. All other circuit components are same as described in Fig. 4 in the manuscript.

a higher disorder strength of $W = 1.5$, we refer to Figs. 1(c), (d), and (e) for the 1st and 52nd, and 100th eigenmodes, respectively. Remarkably, we observe similar features in the TPI behavior compared to the pristine case. The TPI exhibits oscillatory behavior across the nodes, indicating the spatial distribution of the eigenmodes in the presence of disorder. This behavior is consistent with what is typically observed in the local probability density or local IPR of a 1D tight-binding chain without disorder.

However, as we increase the disorder strength further into the localized regime, such as for $W = 2.5$, the behavior of TPI becomes distinct. In Figs. 1(d), (e), and (f), we present the results for the 1st and 52nd, and 100th eigenmodes, respectively. Notably, the TPI values are approximately one order of magnitude larger than those in the delocalized regime. Moreover, the TPI becomes pinned at a specific node, indicating localization of the eigenmodes. This behavior is strikingly similar to what is observed in localized states in a 1D tight-binding chain.

Based on these distinguishable features of the TPI, we can unambiguously refer to the localization transition in the circuit. The TPI values and their characteristic behaviors serve as reliable indicators of whether the circuit is in a delocalized or localized regime, providing valuable insights into the localization properties of the eigenmodes as the disorder strength varies.

References

[1] S. M. Rafi-Ul-Islam , Z. B. Siu, and M. B. A. Jalil, *Topological phases with higher winding numbers in nonreciprocal one-dimensional topolectrical circuits*, Phys. Rev. B **103**, 035420 (2021).
